# Challenges in the care of individuals with severe primary insulin-like growth factor-I deficiency (SPIGFD): an international, multi-stakeholder perspective

**DOI:** 10.1186/s13023-023-02928-7

**Published:** 2023-10-07

**Authors:** Philippe F. Backeljauw, Mary Andrews, Peter Bang, Leo Dalle Molle, Cheri L. Deal, Jamie Harvey, Shirley Langham, Elżbieta Petriczko, Michel Polak, Helen L. Storr, Mehul T. Dattani

**Affiliations:** 1 Cincinnati Children’s Hospital Medical Center, University of Cincinnati College of Medicine, Cincinnati, OH USA; 2 The Major Aspects of Growth in Children (MAGIC) Foundation, Warrenville, IL USA; 3 The MAGIC Foundation International Coalition for Organizations Supporting Endocrine Patients (MAGIC-ICOSEP), Atlanta, GA USA; 4 Division of Children’s and Women’s Health, Department of Biomedical and Clinical Sciences (BKV), Faculty of Health Sciences, Linköping University, Linköping, Sweden; 5 Caregiver Representative, Cincinnati, OH USA; 6 Université de Montréal, Montréal, QC Canada; 7 Centre Hospitalier Universitaire (CHU) Sainte-Justine, Montréal, QC Canada; 8 Paediatric Endocrinology, Great Ormond Street Hospital UCL Hospitals, London, UK; 9 Department of Paediatrics, Endocrinology, Diabetology, Metabolic Disorders, and Cardiology of Developmental Age, Pomeranian Medical University, Szczecin, Poland; 10 Department of Pediatric Endocrinology, Gynecology and Diabetology, Hôpital Universitaire Necker Enfants Malades, Assistance Publique Hôpitaux de Paris, Université Paris Cité, Paris, France; 11 Centre for Endocrinology, William Harvey Research Institute, Barts and the London School of Medicine and Dentistry, Queen Mary University of London, London, UK; 12 UCL Great Ormond Street Institute of Child Health, London, UK; 13 Adolescent Endocrinology, UCL Hospitals, London, UK

**Keywords:** Insulin-like growth factor-I (IGF-I), Severe primary IGF-I deficiency (SPIGFD), Growth hormone insensitivity (GHI), Short stature, Laron syndrome, Diagnosis, Treatment, Quality of life

## Abstract

**Background:**

Severe primary insulin-like growth factor-I (IGF-I) deficiency (SPIGFD) is a rare growth disorder characterized by short stature (standard deviation score [SDS] ≤ 3.0), low circulating concentrations of IGF-I (SDS ≤ 3.0), and normal or elevated concentrations of growth hormone (GH). Laron syndrome is the best characterized form of SPIGFD, caused by a defect in the GH receptor (*GHR*) gene. However, awareness of SPIGFD remains low, and individuals living with SPIGFD continue to face challenges associated with diagnosis, treatment and care.

**Objective:**

To gather perspectives on the key challenges for individuals and families living with SPIGFD through a multi-stakeholder approach. By highlighting critical gaps in the awareness, diagnosis, and management of SPIGFD, this report aims to provide recommendations to improve care for people affected by SPIGFD globally.

**Methods:**

An international group of clinical experts, researchers, and patient and caregiver representatives from the SPIGFD community participated in a virtual, half-day meeting to discuss key unmet needs and opportunities to improve the care of people living with SPIGFD.

**Results:**

As a rare disorder, limited awareness and understanding of SPIGFD amongst healthcare professionals (HCPs) poses significant challenges in the diagnosis and treatment of those affected. Patients often face difficulties associated with receiving a formal diagnosis, delayed treatment initiation and limited access to appropriate therapy. This has a considerable impact on the physical health and quality of life for patients, highlighting a need for more education and clearer guidance for HCPs. Support from patient advocacy groups is valuable in helping patients and their families to find appropriate care. However, there remains a need to better understand the burden that SPIGFD has on individuals beyond height, including the impact on physical, emotional, and social wellbeing.

**Conclusions:**

To address the challenges faced by individuals and families affected by SPIGFD, greater awareness of SPIGFD is needed within the healthcare community, and a consensus on best practice in the care of individuals affected by this condition. Continued efforts are also needed at a global level to challenge existing perceptions around SPIGFD, and identify solutions that promote equitable access to appropriate care.

Medical writing support was industry-sponsored.

**Supplementary Information:**

The online version contains supplementary material available at 10.1186/s13023-023-02928-7.

## Introduction

**Fig. 1:**
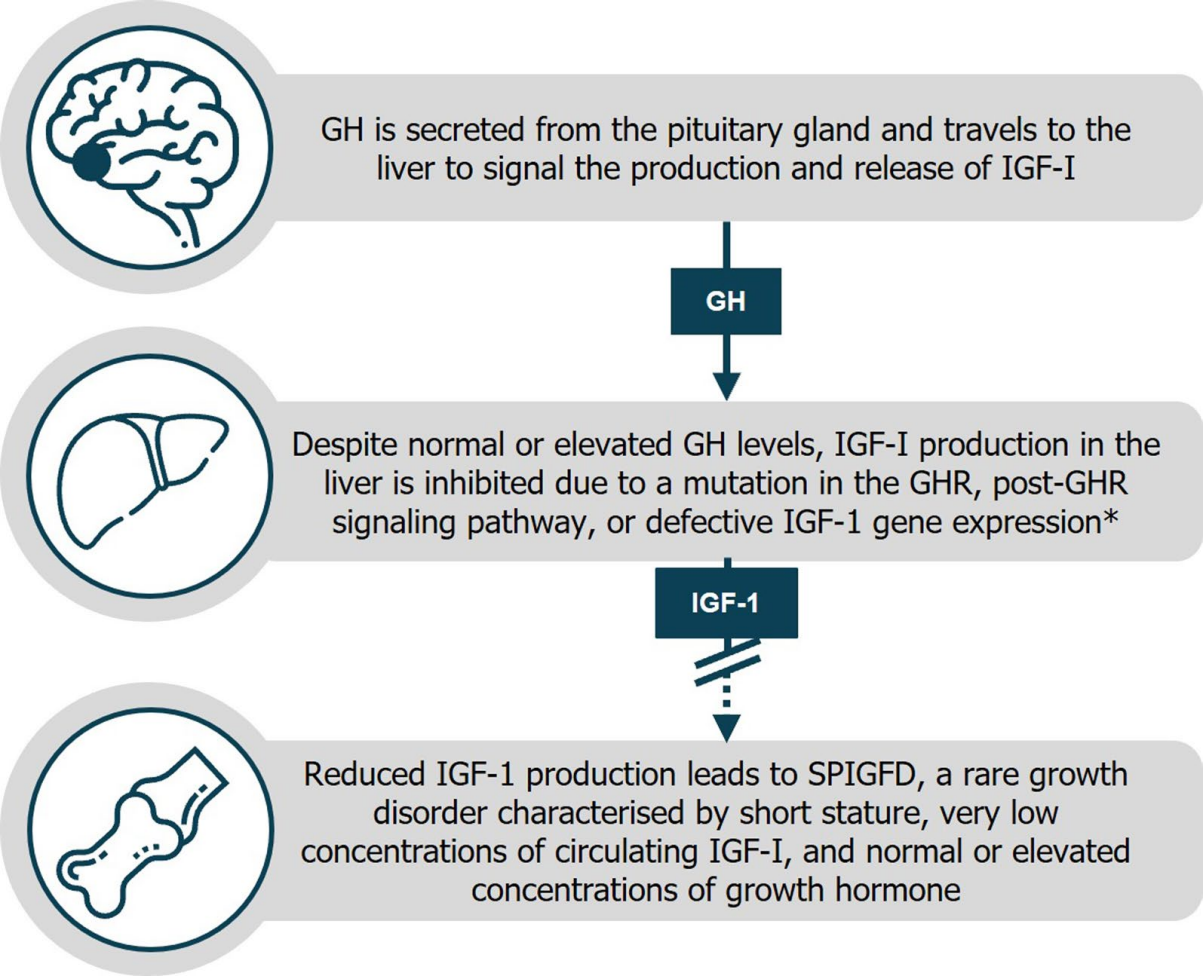
SPIGFD disease pathway. *The liver is the main producer of circulating IGF-I; however, GH-stimulated IGF-I production also occurs in multiple tissues. GH, growth hormone; GHR, growth hormone receptor; IGF-I, insulin-like growth factor; SPIGFD, severe primary IGF-I deficiency

Severe primary insulin-like growth factor-I (IGF-I^1^) deficiency (SPIGFD) is a rare growth disorder which falls under the wider spectrum of IGF-I deficiency [1–3]. SPIGFD is characterized by severe short stature, low concentrations of circulating IGF-I, and normal or elevated concentrations of growth hormone (GH) [4]. A simple overview of the SPIGFD disease pathway is presented in Fig. 1; further details of the molecular pathways involved in IGF-I signaling have been described elsewhere (for example, Puch and Castilla-Cortázar [2012]; Argente et al. [2017]) [5, 6]. The global prevalence of SPIGFD is uncertain, in part due to varied definitions of the condition [2, 4, 7]. In the European Union (EU), it is estimated that 2 per 10,000 people are affected by primary IGF-I deficiencies (PIGFD) [8, 9]; as a subcategory of PIGFD, it is assumed that SPIGFD affects even fewer individuals. Approximately 0.8–1.2% of children who are referred to a pediatric endocrinologist for suspected slow statural growth are diagnosed with SPIGFD, based on a French cohort study [2]. Recombinant human IGF-1 (rhIGF-1) is currently the only effective therapy for patients with SPIGFD [7].

SPIGFD can be caused by mutations in key genes involved in the GH-IGF axis, such as those encoding the GH receptor (GHR), signal transducer and activator of transcription 5B (STAT5B), IGF-I or acid-labile subunit (ALS) (Table 1) [10, 11]. The best characterized, “classic” form of SPIGFD is Laron syndrome, caused by mutations in the *GHR* gene [12, 13]. European registry data highlight that there are likely various genetic causes of IGF-I deficiency which remain poorly characterized, with a confirmed *GHR* deletion or mutation accounting for only 13% of children receiving rhIGF-1 enrolled in the EU Increlex® Growth Forum Database registry [14]. Additionally, milder forms of the condition may remain undetected [12]. Thus, the diagnostic pathway for SPIGFD is challenging at present and the exact genotype distribution remains uncertain.

**Table 1:** Summary of known genetic causes of SPIGFD

Affected gene	Details
*IGFALS*	Decreased serum ALS as a result of a defect in the *IGFALS* gene is associated with moderate short stature, delayed puberty and decreased serum IGF-I
*GHR*	Genetic defects in the *GHR* gene are the underlying cause of the classic form of SPIGFD, known as Laron syndrome
*IGF-I*	Homozygous mutations in *IGF-I* are very rare and are associated with severe short stature, deafness and insulin resistance
*IκBα*	*IκBα* mutations are associated with short stature and immunodeficiency
*PAPPA2*	Defects in *PAPPA2* disrupt the release of circulating IGF-I, leading to varying degrees of short stature and insulin resistance
*PTPN11*	Activating *PTPN11* mutations leads to dephosphorylation of *STAT5B*, causing downregulation of its activity and partial GH insensitivity
*STAT5B*	Mutations in *STAT5B* can lead to short stature, as well as a severe immune dysfunction

*Source*: Wit et al. 2012 [10]; Hwa et al. 2021 [11]

GHR, growth hormone receptor; IGFALS, insulin-like growth factor acid labile subunit; IGF-I, insulin-like growth factor I; IκBα, nuclear factor of kappa light polypeptide gene enhancer in B-cells inhibitor, alpha; PAPPA2, human pregnancy-associated plasma protease A2; PTPN11, tyrosine-protein phosphatase non-receptor type 11; SPIGFD, severe primary IGF-I deficiency; STAT5B, signal transducer and activator of transcription 5B

Evidence from a large Israeli cohort suggests that SPIGFD places a significant burden on individuals and their caregivers, including challenges navigating the diagnostic pathway, as well as practical difficulties in terms of schooling, mobility, obtaining age-appropriate clothing of a suitable size, and providing an ergonomic environment as individuals enter adulthood [15]. However, the non-growth-related effects of SPIGFD are largely undefined, and the overall impact of the condition on patients and their families on a global scale is poorly understood. To gather perspectives around the wider challenges faced by patients with SPIGFD, a multi-stakeholder meeting was convened, bringing together an international panel of clinical experts, researchers, caregivers, and representatives from patient advocacy organizations. Drawing on their expertise and/or lived experience, the group identified key challenges and highlighted opportunities and recommendations to help address unmet needs and improve the care of people living with SPIGFD. Key outcomes from the discussions are presented here.

## Methods

A targeted literature review was conducted to identify evidence on the diagnosis, access to treatment, unmet needs, and best practices in SPIGFD care. Literature on the humanistic, societal, and economic burden of SPIGFD was also reviewed. The evidence gathered was used to inform discussion topics for the multi-stakeholder meeting.

The virtual, half-day meeting had a total of 11 participants and was comprised of clinicians, a clinical nurse specialist, researchers, parents of children with SPIGFD, and representatives from patient advocacy organizations. The group included a number of internationally renowned experts, and provided perspectives from six countries (Canada, France, Poland, Sweden, the United Kingdom [UK] and the United States [US]). Two experts in the field (Philippe Backeljauw and Mehul Dattani) co-chaired the meeting.

Consistent with best practice reporting of patient involvement in research, patient and public involvement in this project is reported using the standardized Guidance for Reporting Involvement of Patients and the Public (GRIPP) checklist (Additional file 1) [16].

## Awareness

**Fig. 2:**
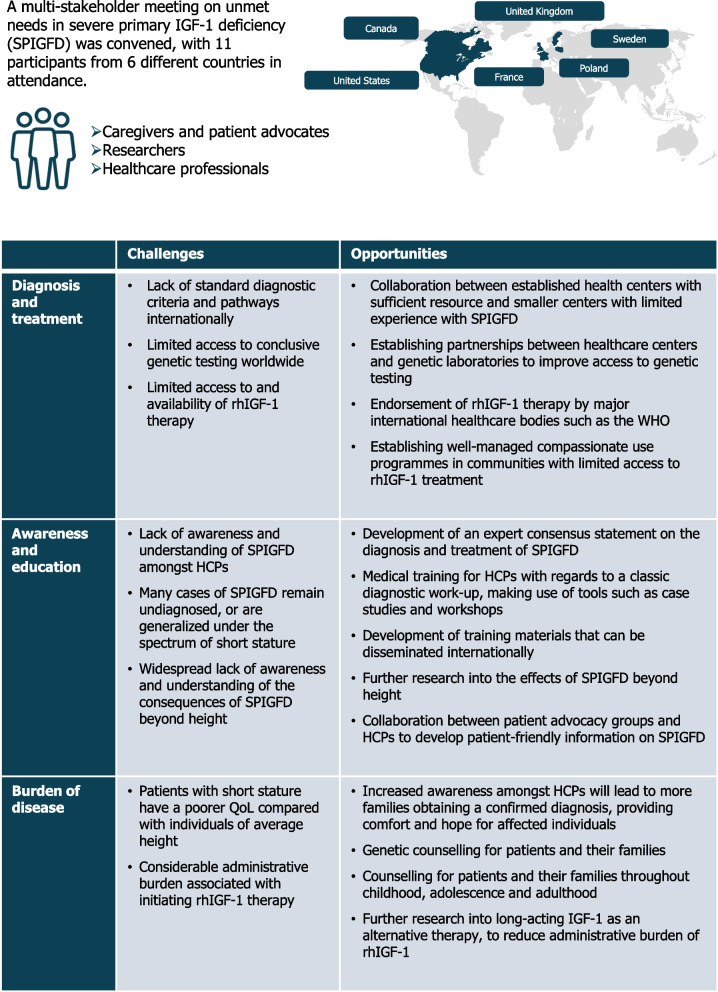
Summary of key challenges and opportunities identified during the multi-stakeholder meeting. HCP, healthcare professional; IGF-I, insulin-like growth factor I; QoL, quality of life; rhIGF-1, recombinant human IGF-I; SPIGFD, severe primary IGF-I deficiency; WHO, World Health Organization

The key challenges and opportunities identified during the multi-stakeholder meeting are summarized in Fig. 2. It is estimated that the global point prevalence of rare diseases combined is 3.5–5.9%, affecting a total of 263–446 million individuals at any given time [17]. However, the knowledge and awareness of specific rare diseases amongst healthcare professionals (HCPs) and wider communities remains inadequate. For example, recent studies suggest that training and education of rare diseases amongst HCPs is limited, with one study reporting that as many as 94.6% of clinicians perceived their knowledge to be poor [18–20]. As a rare disorder, there is a general lack of awareness around SPIGFD within healthcare and wider communities [21]. Very few HCPs have first-hand experience of caring for patients with SPIGFD, and newly-appointed pediatric endocrinologists may lack the necessary exposure to recognize the condition. In addition, much of the research and dissemination of information around IGF-I deficiency focuses on the genetic particulars of diagnosis and treatment [11, 22–24], rather than the practicalities of these processes. This has significant implications for the diagnosis and treatment of SPIGFD, with primary HCPs unequipped to properly diagnose the condition and refer patients to specialists. The introduction of dedicated medical education symposia, focusing on the practicalities of diagnosis and treatment, would improve the awareness of SPIGFD and provide a platform to address the unmet needs in SPIGFD care.

The general lack of awareness and understanding of SPIGFD also permeates more widely, with limited information targeted at those outside of the medical community. Access to resources that offer a holistic understanding of SPIGFD is particularly important for patients and their families, providing the tools to understand the complexities of SPIGFD and emphasize the importance of early diagnosis and treatment adherence. Patient and caregiver advocacy groups, such as The MAGIC Foundation (US) [25], and the Child Growth Foundation (UK) [26], play key roles in this respect, providing platforms for families to share their experiences and concerns via a network of social media outlets and conferences. These groups also offer valuable support through educational materials for patients with growth disorders. However, there is an ongoing need for readily accessible information to raise awareness and understanding of SPIGFD among those affected by the condition and the wider community.

## Diagnosis

### Clinical diagnosis

To diagnose SPIGFD, HCPs refer to a broad range of diagnostic criteria and tests, such as those relating to growth measurements. However, there are differences in published diagnostic pathways, and varied clinical criteria for SPIGFD diagnosis across geographies [27]. For instance, due to differences in national growth charts the diagnostic threshold of 3 SDS translates into different values for measured height depending on the country-specific context. This results in potential variations in diagnostic criteria and SPIGFD thresholds on a global scale. For example, growth charts across France, Sweden and Italy specify a height of 3 SDS for a 5-year old male to be 97.0 cm, 98.0 cm and 95.5 cm, respectively [28–30]. The use of geographically-appropriate and up-to-date growth charts is therefore important. Further, some growth charts only provide percentiles, which require pediatric endocrinologists to manually calculate SDS for children with suspected SPIGFD, making comparison more challenging.

As part of a spectrum of disorders with varying degrees of severity, the perceived narrow margin for the diagnosis of SPIGFD can potentially restrict HCPs from giving consideration to less “classic” types of SPIGFD, including some autosomal dominant forms. This can delay diagnosis and therefore treatment initiation, which can impact treatment outcomes. In cases of familial short stature, where short stature is inherited from one or both parents, it is imperative that a full diagnostic work-up is carried out to not only identify chronic, unrecognized illness but also confirm any possible cases of SPIGFD [31, 32]. Similarly, it is important that further IGF-I measurements are carried out in children that exhibit a poor response to stepwise titration of GH treatment [33].

### Biochemical diagnosis

Biochemical criteria are often necessary to support a comprehensive SPIGFD diagnosis in this heterogeneous patient population [27, 34]. Testing for IGF-I deficiency typically involves the measurement of serum IGF-I and demonstration of normal or increased GH secretion [27, 31]. However, concerns have been raised regarding the sensitivity of commercially available-assays in measuring IGF-I concentrations, which are dependent on a range of factors including age, sex, pubertal stage, nutritional status and genetic factors [2, 27].

Additionally, there is a lack of normative data in terms of IGF-I SDS and percentiles, and data for the youngest pediatric patients are limited, resulting in a wide "normal" range and making a biochemical diagnosis of SPIGFD more difficult. While published recommendations for biochemical assays are available [35], they appear to have had limited uptake by clinicians, resulting in a lack of international standards regarding sample collection/storage procedures. This has led to variability, both in terms of the estimated values and clinical interpretation of hormone concentration across different assays [2, 36]. In addition, the diagnostic criteria relating to IGF-I serum concentrations also vary across different geographies. For example, the basal IGF-I concentration threshold for SPIGFD diagnosis is SDS ≤ 3.0 in the US, but < 2.5th percentile in the EU [27].

Although not a requirement for SPIGFD diagnosis, the IGF-I generation test also can be used in clinical practice to investigate GH secretion and sensitivity, therefore supporting the diagnostic process [31]. However, a lack normative reference data means that the results of this test are seldom clear-cut, except in cases of classic SPIGFD where extreme GH resistance is usually present [37, 38]. As such, conducting this test in non-classic cases of SPIGFD can result in prolonged and unnecessary investigations, associated with significant economic and resource burden on healthcare services [39].

### Genetic diagnosis

First-line genetic testing to confirm a diagnosis of SPIGFD typically involves sequencing of the *GHR* gene or other genes involved in the GH-IGF axis (Table 1) [37]. However, additional genetic analyses may be required, particularly in non-classic cases of IGF-I deficiency, as it can provide conclusive results, therefore avoiding unnecessary diagnostic investigations and facilitating earlier treatment initiation [40]. A number of commercially available gene panels, from companies such as Blueprint Genetics®, Fulgent® and GHC Genetics® now offer a conclusive genetic diagnosis [41–43]. However, having a genetic diagnosis is not a prerequisite for receiving rhIGF-1 treatment, and does not affect response outcomes, as demonstrated by EU-IGFD data [44, 45].

Unfortunately, genetic testing is associated with high costs, and there are limited numbers of laboratories with the necessary equipment and specialist knowledge to conduct this type of genetic analysis [40]. As such, access to genetic testing to support SPIGFD diagnosis is limited in many regions; particularly for patients in developing countries and from lower-income backgrounds, genetic testing may not be an option. Countries with particularly poor access to genetic testing often rely on outsourcing to overseas laboratories, prolonging the time to diagnosis for patients in these regions [46]. Establishing partnerships between healthcare centers and genetic laboratories, and encouraging increased collaboration between centers with genetic testing resources and smaller centers with limited experience in SPIGFD, would provide an opportunity to improve diagnosis for patients. These robust referral networks would also likely increase broader awareness of SPIGFD amongst HCPs.

## Access to treatment

Once a diagnosis of SPIGFD has been established, rhIGF-1 therapy should commence as soon as possible [4]. However, discussions highlighted that many individuals receive treatment at unacceptably late stages due to delayed or missed diagnoses, both of which are exacerbated by limited awareness of SPIGFD among HCPs. Given that a continuous loss of height SDS occurs with increased age for affected individuals, those that fall below a height of 3 SDS early on ultimately experience a more severe degree of short stature than those in whom this occurs at an older age [44]. Early intervention is therefore particularly important for children with SPIGFD, since it optimizes long-term treatment outcomes and thus reduces the burden of short stature into adulthood [47]. Findings from EU-IGFD registry data suggest that treatment initiation for treatment-naïve pre-pubertal patients occurs at a mean (SD) age of 6.07 (3.49) years for individuals with Laron syndrome and 8.44 (3.45) years for those with uncharacterized forms of IGFD [44]. The registry findings indicate that earlier treatment initiation is the only significant predictive factor of response to rhIGF-1 therapy (0.13 [95% CI by 1 unit increment: 0.25; 0.01]; *p* < 0.033), and that prepubertal patients naïve to treatment were able to achieve a mean height velocity during the first year of rhIGF-1 treatment of 7.3 cm/year (95% CI 6.8, 7.7) (n = 81) [14].

Access to rhIGF-1 differs across geographic regions, depending on the regulatory, political, and wider socio-economic environment. Despite being developed in 1986, rhIGF-1 was only authorized for use in the US by the Food and Drug Administration (FDA) in 2005, without specific coding available for its prescription until 2021 [48]. In Europe, the treatment was granted marketing authorization under exceptional circumstances by the European Medicines Agency (EMA) in 2007 [1, 49]. In Canada, rhIGF-1 was only approved by Health Canada in 2021 [50]. In some areas where rhIGF-1 is not licensed, such as Australia, patients may seek therapy through managed access programs [51]. Yet for other regions, including most of South America, the Middle East and Africa, people with SPIGFD are unable to access rhIGF-1 locally due to a lack of approval. Poor infrastructure, political unrest, economic challenges, or other barriers to patient safety follow-up can further hinder access for these patients, and lead managed access programs that do exist in these areas to be withdrawn or suspended unexpectedly [46]. Such challenges are relevant to countries in the Middle East, where SPIGFD cohorts have been well-characterized but many patients are from low socio-economic backgrounds [46].

To encourage the approval of SPIGFD treatments around the world, there is an unmet need to improve the understanding of SPIGFD epidemiology and treatment response, particularly in regions where SPIGFD is less well-characterized. The increased use of patient registries for data collection could facilitate such efforts. Endorsement of rhIGF-1 from major international healthcare agencies, such as the World Health Organization (WHO), could also support broader authorization and approval. Meanwhile, to improve access for SPIGFD communities in regions where treatments are not locally available, the introduction of well-managed compassionate use programs could help to address the challenge of inequitable access.

## Medical education

The identification and diagnosis of patients with SPIGFD is an active process, requiring specific training and expertise amongst HCPs [27]. In practice, however, HCPs tend to lack the necessary understanding to recognize the classic features of Laron syndrome, let alone the non-classic phenotypes of IGF-I deficiency (e.g. heterozygous *STAT5B* mutations) [52]. As a result, many cases of SPIGFD either remain undiagnosed or are generalized under the wider spectrum of idiopathic short stature.

In order to improve the clinical diagnosis, specific education and exposure of HCPs (including primary care physicians and pediatric endocrinologists) to a classic diagnostic work-up for short stature, and in particular for SPIGFD, is recommended. This should include a detailed patient history and information on appropriate testing. In addition, HCPs should be offered training to identify the classic SPIGFD phenotypes (Table 2) and genotypes (Table 1), as this would likely facilitate improved understanding of non-classic and less severe cases of IGF-I deficiency. Training courses or programs targeting junior endocrinologists, in particular, would provide the necessary background knowledge to appropriately diagnose patients with IGF-I deficiency. Training materials for HCPs should be made available in multiple languages to ensure equitable accessibility and dissemination of resources internationally.

**Table 2:** Table of known phenotypic and non-growth characteristics of SPIGFD

Clinical features	Details
Growth	Normal birth weight and length
	Growth failure from birth
	Height deviation which correlates with low serum IGF-I
	Delayed bone age
	Small hands and/or feet
Craniofacial characteristics	Sparse hair before age 7; fronto-temporal hairline recession at all ages
	Prominent forehead
	Disproportionally large head due to small stature
	“Setting sun sign” (sclera visible above iris at rest) in 25% of patients under the age of 10 years
	Hypoplastic nasal bridge with shallow orbits
	Decreased vertical dimension of face
	Blue sclerae
	Prolonged retention of primary dentition with decal; normal permanent teeth with crowding
	Unilateral ptosis, facial asymmetry
Musculoskeletal composition	Decreased muscle mass with delay in walking
	Avascular necrosis of femoral head (25%)
	High pitched voice
	Thin, prematurely aged skin
	Limited elbow extensibility after 5 years of age
	Low to normal BMI during childhood, developing into high BMI in adulthood
	Markedly decreased ratio of lean mass to fat mass compared to normal
Neurocognition	Impact on brain growth and structure
	Reduced cognitive function and motor performance
Metabolic characteristics	Hypoglycemia
	Increased cholesterol and LDL-C
	Decreased sweating
	Insulin resistance
Sexual development	Small genitalia in males during childhood
	Delayed puberty
	Normal reproduction
Other features	Deafness
	Immunodeficiency or severe immune dysfunction

*Source*: Guevara-Aguirre et al. 1991 [55]; Laron 2001 [56]; Webb et al. 2012 [57]; Higashi et al. 2019 [58]; Cohen et al. 2014 [4]; Backeljauw et al. 2010 [27]; Bang et al. 2012 [37]; Ascenzi et al. 2019 [59]

This list of non-growth effects of SPIGFD is non-exhaustive; there is currently limited research available on the non-growth effects of SPIGFD available and these remain poorly understood

BMI, body mass index; IGF-I, insulin-like growth factor I; LDL-C, low-density lipoprotein cholesterol; SPIGFD, severe primary IGF-I deficiency

Drawing connections between SPIGFD and other phenotypically similar short stature syndromes associated with IGF-I deficiency or partial GH insensitivity, such as Silver-Russell syndrome or Noonan syndrome, would provide a good starting point to build awareness and understanding around this rare condition within the medical community [8]. For example, typical clinical features of Noonan syndrome include short stature that may or may not be associated with low IGF-I concentrations, craniofacial characteristics and cardiac defects that may lead to a misdiagnosis of SPIGFD, or vice versa. However, unlike for patients with SPIGFD, the first-line treatment recommended for patients with Noonan syndrome is recombinant human growth hormone (rhGH) [53, 54]. In addition to further research being necessary to better characterize the spectrum of short stature syndromes, there is also a need for further training to support HCPs to recognise key clinical nuances between such disorders in order to ensure a timely and accurate diagnosis.

There is also a need for HCP training on the expected benefits of rhIGF-1 therapy for SPIGFD. Because first year height velocity data suggests that the impact of rhIGF-1 among SPIGFD patients is less pronounced than rhGH for GH-deficiency (rhIGF-1: 6.9 [6.5, 7.2] cm/year [n = 144] vs rhGH: 8.67 [7.5, 9.9] cm/year [n = 465]) [14, 60], some HCPs may not perceive rhIGF-1 as beneficial enough for SPIGFD. However, this should not constitute a reason to disregard treatment; an increase to height velocity of 6.9 cm/year can still have meaningful benefits to SPIGFD patients. Training to communicate this to HCPs would be valuable. Such training should also look to provide reassurance to HCPs that the potential benefits of rhIGF-1 often outweigh the risk of side effects; in particular, of hypoglycemic events. Hypoglycemia is the most frequent adverse event (AE) associated with untreated IGF-I deficiency which, without suitable management, can worsen with rhIGF-1 treatment; estimates from studies in Poland and the US suggest that 7–42% of rhIGF-1 treated patients with SPIGFD experience hypoglycemia [12, 61]. This may cause hesitancy amongst HCPs when prescribing rhIGF-1 [14, 37, 44, 62]. However, other studies have shown that frequency of hypoglycemic events does not increase with rhIGF-1 therapy, and effective prevention of glycemic decline can be maintained through proper nutrition and monitoring [62–64]. Further, the frequency of hypoglycemia AEs reported in clinical trials (49%) is notably higher than in post-marketing data (28%), suggesting that this risk can be managed through adequate glucose monitoring [4, 14, 65, 66]. Capillary blood glucose monitoring upon initiation of rhIGF-1 therapy is recommended until a well-tolerated dose is established [31]. The use of case studies for children who have undergone rhIGF-1 therapy, including data on adult height and hypoglycemic events, could be an important educational tool for HCPs to share experience and best practice.

## Perspectives on SPIGFD

The primary objective of rhIGF-1 therapy is to achieve an adult height within the target range for the individual [31]. However, SPIGFD may also affect mobility and have a negative impact on everyday activities. For example, individuals of short stature may be unable to drive a car or apply for certain occupations, such as becoming a flight attendant which has a minimum height restriction of 4 feet 11 inches [67]. Evidence also suggests that IGF-I is important for cellular growth, heart and lung function, thereby reducing the risk of cardiac arrest and acute lung injury [58, 68–70]. As such, low circulating concentrations of IGF-I may have serious health implications on patients with SPIGFD, beyond height.

Unfortunately, the lack of understanding and limited investigation into the non-height-related effects of IGF-I deficiency (Table 2) has led to a misconception of SPIGFD as a predominantly cosmetic, height-related disorder. This, in turn, is likely to have contributed towards the general oversight of SPIGFD at a global level and awareness of the tangible impacts that the condition has on patient wellbeing and everyday activities. By bringing the non-height-related consequences of IGF-I deficiency to light, it may be possible to generate a wider interest in SPIGFD from an industry and policymaker perspective.

Sadly, children with short stature are often juvenilized by their caregivers and teachers, and short-stature adults are often branded and perceived to have diminished mental capacity, both in a social and clinical setting. The GH-IGF axis plays a role in normal brain growth and structure, and severe GH deficiency has been associated with altered neurocognition in some patients (possibly related to frequent episodes of hypoglycemia) [57]. However, the normalization of pejorative terms through academic journals and discussions enforces the inaccurate notion that individuals with short stature are "abnormal" and leads to misconceptions around the intelligence of patients with SPIGFD [39]. It is imperative to overcome this misunderstanding of patients with short stature to improve care for patients with SPIGFD. As such, efforts should be made in the medical field, as well as schools and workplaces, to abolish derogatory terms and assumptions around patients with SPIGFD and other short stature conditions.

## Burden of disease

Research into quality of life for children with short stature suggests that they suffer from low self-esteem and poor concentration compared with children of average height [71]. Short stature children often experience bullying and discrimination in the school environment due to their height; this can continue into adulthood, potentially impacting their ability to find a life partner or employment [15, 46, 72]. In developing countries, in particular, this can have a marked impact on patients’ ability to make a living and provide food for themselves and their families [46]. Studies have also demonstrated that short stature can impact the psychological and social wellbeing of people living with SPIGFD [39]. It is not uncommon for individuals to experience emotional suffering and depression due to the social and economic challenges that affect their daily lives [72]. It is imperative that those affected by SPIGFD have access to the necessary support and counselling, throughout childhood, puberty and into adulthood.

The period before and during diagnosis can be particularly challenging for patients and their families. Some parents may blame themselves, wondering what they could have done differently. Parents may even live in fear that their child will be unable to lead an independent life and concerns regarding the impact of short stature on psychosocial function are a key influencing factor in parental motivation to seek medical care [73]. The absence of information and understanding can lead to uncertainty and anxiety amongst affected families trying to obtain a clear diagnosis and access treatment for their child. As an experience faced by many living with rare conditions, a prolonged process of referrals can be distressing, particularly for young children and their families [74]. Increased awareness around this disorder would mean that patients receive a timely and formal diagnosis, along with adequate genetic counselling, providing comfort and hope for the future [15].

Treatment initiation can also be a burdensome step for SPIGFD patients and their families, with the commencement of treatment sometimes having a significant time and economic cost [75]. For example, UK clinicians often see patients admitted to hospital and allocated a period of ~ 5 days to initiate treatment if blood glucose control is anticipated to be problematic, with additional home support provided, resource permitting. Contrastingly, in the US and other European countries such as Poland and Sweden, the initiation of treatment proceeds in an outpatient setting. Aside from cost, the benefit of commencing treatment in the outpatient setting is that it signals to patients and their families that the condition is easily manageable, without need for hospitalization. However, the disadvantage to this approach is that patients may be required to attend multiple visits, conferring additional financial and time costs to patients and their families. Additionally, occurrences of hypoglycemic events may not be detected as easily in these children, though regular capillary blood glucose measurements by patients or their caregivers may help to monitor such occurrences.

Following a confirmed diagnosis, the initiation of treatment itself requires substantial resource and instruction for caregivers in terms of home administration, blood glucose monitoring and management of injection site reactions. Given the onerous requirement to administer two subcutaneous injections per day, treatment with rhIGF-1 can put a strain on family activities, with panel members reporting that it is not uncommon for siblings to receive less parental attention than children with SPIGFD. Given this burden, it is important that families are aware of the expectations and requirements of treatment before initiation. HCPs should explain to caregivers that maintaining treatment adherence is critical to improving growth for children with SPIGFD, with one study demonstrating that rhIGF-1 resulted in an overall height gain of 13.4 cm more than expected without treatment for SPIGFD patients, with 10.0 years of follow-up (n = 21) [62]. Clearly communicating the requirement and expected long-term benefits are thus important in ensuring that parents (and subsequently, patients themselves) are well-supported to administer the therapy.

## Future directions

Based on the outcomes of the multi-stakeholder meeting (Fig. 2), it is clear that targeting the right audience is fundamental in improving diagnosis and treatment of SPIGFD. Specifically, raising awareness amongst primary HCPs should be a priority, since improving the awareness and understanding of SPIGFD amongst the healthcare community would likely provide a framework to improve access to information for those directly affected by SPIGFD, and wider society as a whole.

The development of an updated expert consensus statement on the diagnosis and treatment of SPIGFD could provide much needed guidance for HCPs. Clear guidance on the most appropriate diagnostic work-up is imperative to give HCPs the confidence to initiate treatment or refer patients to specialists in a timely manner. Similarly, an algorithm on the diagnosis and management of IGF-I deficiency would provide a useful resource, illustrating the potential for various diagnostic tools to characterize SPIGFD and other forms of IGF-I deficiency. There is also a need to better characterize the complexities and treatment response of IGF-I deficiency worldwide by gathering more data through patient registries. The development of such materials would benefit from the support of international organizations committed to advancing research and education relating to growth disorders. This, in combination with well-developed compassionate use programs and the dissemination of accessible information, would foster a better understanding of the landscape of IGF-I deficiency disorders internationally, and contribute towards improved diagnosis, access to treatment, and burden of care for patients worldwide.

Evidently, there is a need to challenge current perceptions around SPIGFD. Recognizing the physical, emotional, and social impacts of SPIGFD on patients and their families is a good starting point. Support and counselling should be offered to children with SPIGFD, and maintained throughout puberty, higher education and adulthood [15]. Moreover, shifting the goal of SPIGFD treatment from making children taller to making them healthier will be key to improving awareness and access to care for patients with SPIGFD.

## Conclusions

The outcomes of the multi-stakeholder meeting summarized in this paper highlight the challenges faced by patients, families and HCPs around SPIGFD. As a rare endocrine disorder with an indistinct diagnostic and treatment pathway, awareness of this condition is limited, with diagnosis (and, subsequently, treatment) often delayed. Panel members emphasized the need to educate and provide clearer guidance to HCPs to facilitate earlier diagnosis and initiation of appropriate treatment, with an ultimate goal of improving clinical outcomes and quality of life for those living with SPIGFD. For regions where access to diagnostics and appropriate treatment is limited due to poor infrastructure, political unrest, regional conflicts and/or economic barriers, efforts to identify solutions to promote equitable access to appropriate pathways to care are particularly important.

Key recommendations from the multi-stakeholder meeting included the development of widely accessible materials to educate and challenge existing perceptions around SPIGFD to shift focus away from height as the overarching measurable outcome of the condition and provide valuable guidance and support. Furthermore, continued efforts from clinicians, patient advocacy groups, medical societies, and other key stakeholder groups to enhance awareness and understanding of this complex condition on a global level will be vital to address many of the challenges raised in this paper and improve the holistic care for patients living with SPIGFD.

### Supplementary Information

**Additional file 1**: Title of data: GRIPP2 table of patient and public involvement*. Description: Table presenting the GRIPP2 checklist, reporting patient and public involvement.

## Data Availability

Not applicable.

## Footnotes

1 IGF-I is used to describe the peptide; IGF-1 is used to describe the pharmaceutical agent.
