# Supplementary material for: Challenges in the care of individuals with severe primary insulin-like growth factor-I deficiency (SPIGFD): an international, multi-stakeholder perspective
Source: Orphanet J Rare Dis. 2023 Oct 7;18:312. doi: 10.1186/s13023-023-02928-7 (PMC10559630; doi:10.1186/s13023-023-02928-7)
Supplement: Supplementary file 1 — Additional file 1: Title of data: GRIPP2 table of patient and public involvement*. Description: Table presenting the GRIPP2 checklist, reporting patient and public involvement. [file 13023_2023_2928_MOESM1_ESM.docx]

Additional File 1. GRIPP2 table of patient and public involvement*

| **Section and Topic** | **Item** |
| --- | --- |
| 1: Aim | - To convene a multi-stakeholder meeting, including caregivers and patient representatives, to identify and discuss key challenges, priority areas for change, and opportunities to improve care for people living with severe primary insulin-like growth factor-1 deficiency (SPIGFD) - To develop a shared-voice publication based on these discussions with meeting participants as authors, and to make recommendations for improvement |
| 2: Methods | - Five caregivers, three of which were associated with patient advocacy groups, were invited to participate. Two of the invited caregivers (one individual from a patient advocacy group) did not participate in the project - The caregivers were integral to the discussions during the meeting, which formed the framework and basis for the paper, reviewed and critically appraised multiple drafts of the manuscript and approved the final version for submission |
| 3: Study results | - **Positive**: Throughout the meeting, the caregivers’ ‘real-world’ insight based on personal experience was central to identifying the key challenges and opportunities for people living with SPIGFD. Their input provided valuable context during the development of the manuscript and caregivers felt that the project served as a necessary and important first step in raising awareness of SPIGFD to the wider population - **Learnings:** Dedicated introductory materials may have been beneficial for caregivers with limited experience of the publication process |
| 4: Discussion and conclusions | - Caregivers confirmed that this project helped to address an unmet need for the SPIGFD community. This project would not have gone ahead without their input. Their insight and lived experiences helped shape the discussions in the meeting and the manuscript itself - The landscape of SPIGFD is complex and constantly changing, requiring continuous, timely, and honest input from all relevant stakeholders. Perspectives from patient advocates and caregivers are crucial to this collective discussion, particularly given the pediatric nature of this disorder |
| 5: Reflections/critical perspective | - Input from clinicians was invaluable to identifying caregivers who may be interested in participating in this endeavor - The virtual format of the meeting facilitated the gathering of participants from around the world, who may not have been able to participate without needing to travel |

*Guidelines for reporting the impact of patient and public (GRIPP)2 is a standardized checklist used to report patient and public involvement in health and social care research.
